# Clustering-Based Noise Elimination Scheme for Data Pre-Processing for Deep Learning Classifier in Fingerprint Indoor Positioning System

**DOI:** 10.3390/s21134349

**Published:** 2021-06-25

**Authors:** Shuzhi Liu, Rashmi Sharan Sinha, Seung-Hoon Hwang

**Affiliations:** Division of Electronics and Electrical Engineering, Dongguk University-Seoul, Seoul 04620, Korea; shuzhiliu@dongguk.edu (S.L.); rashmisinha@dongguk.edu (R.S.S.)

**Keywords:** fingerprint-based indoor positioning, clustering, RSSI, CNN

## Abstract

Wi-Fi-based indoor positioning systems have a simple layout and a low cost, and they have gradually become popular in both academia and industry. However, due to the poor stability of Wi-Fi signals, it is difficult to accurately decide the position based on a received signal strength indicator (RSSI) by using a traditional dataset and a deep learning classifier. To overcome this difficulty, we present a clustering-based noise elimination scheme (CNES) for RSSI-based datasets. The scheme facilitates the region-based clustering of RSSIs through density-based spatial clustering of applications with noise. In this scheme, the RSSI-based dataset is preprocessed and noise samples are removed by CNES. This experiment was carried out in a dynamic environment, and we evaluated the lab simulation results of CNES using deep learning classifiers. The results showed that applying CNES to the test database to eliminate noise will increase the success probability of fingerprint location. The lab simulation results show that after using CNES, the average positioning accuracy of margin-zero (zero-meter error), margin-one (two-meter error), and margin-two (four-meter error) in the database increased by 17.78%, 7.24%, and 4.75%, respectively. We evaluated the simulation results with a real time testing experiment, where the result showed that CNES improved the average positioning accuracy to 22.43%, 9.15%, and 5.21% for margin-zero, margin-one, and margin-two error, respectively.

## 1. Introduction

With the increase in demand for location-based services, high-precision indoor positioning for smartphones has acquired importance internationally. While the global positioning system (GPS) can be used for positioning in outdoor environments, the reception of GPS signals is poor indoors. Consequently, indoor positioning is challenging. Scholars at home and abroad have proposed many indoor positioning systems for solving the indoor positioning problem, but problems pertaining to their applicability, stability, and expansion persist. On the basis of technology, indoor positioning methods for smartphones can be classified into wireless-network-based, measurement-based sensor, and vision-based positioning methods [1,2,3]. In particular, wireless network-based positioning methods mainly use Wi-Fi, Bluetooth, etc. [4,5,6], among which Wi-Fi positioning is the most widely used positioning method in the literature. There are two main strategies for positioning using Wi-Fi. One is to use a signal propagation model to determine the received signal strength indicator (RSSI), or a channel state information of the Wi-Fi signal to calculate the distance to the access point (AP) for positioning. Another involves constructing a Wi-Fi fingerprint map and using the current Wi-Fi signal to match the fingerprint map to estimate the position [7,8]. This type of fingerprint recognition has notably promoted the development and the usability of the indoor positioning technology.

Many publications [9,10] have reported indoor positioning technology based on the k-nearest neighbor (kNN). Since APs in the environment are significantly displaced from a certain location, APs in certain locations may not be monitored, and the RSSI vector at each location may not include the signals received by all APs. Therefore, the adjacent reference point (RP) may have a similar RSSI vector. Using the kNN algorithm, all RPs on the wireless map consider identifying the nearest neighbor without taking this phenomenon into account. On the other hand, the neighbors found through the kNN algorithm may be scattered in the environment beyond feasible measurement because the signal attenuation of each AP is not only related to distance, but is also affected by many indoor environmental factors. This leads to the minimum signal distance between the RSSI mark position vector and each RP; this distance is not equal to the minimum physical distance between the actual mark position and the recorded RP position. Considering the limitations of the kNN algorithm in indoor positioning technology, indoor positioning technology based on deep learning is an ideal alternative. A previous study [11] experimentally confirmed the possibility of a convolutional neural network (CNN) applied to complex image classification to improve accuracy, particularly in the classification of complex pictures in a dynamic environment. On this basis, another study [12] innovatively utilized the CNN algorithm as an indoor positioning technology framework. Experiments show that CNN can effectively address the limitations of the kNN algorithm and improve the accuracy of indoor positioning.

Hao et al. [13] proposed a fingerprinting technique based on channel state information (CSI). The CSI information of 25 RPs was collected by one transmitter and one receiver with three links of the 121 subcarriers in each link. The size of each RP was 1.1 m × 0.96 m for all 25 RPs. The density-based spatial clustering of applications with noise (DBSCAN) processing of the CSI data is performed in the offline phase. The noise reduction of the processed dataset was performed with the endpoint-clipping method. This endpoint-clipped dataset was used to train the SVM classifier. The DBSCAN processing of the CSI test data was performed in the online phase. Matching of the test location was done by matching the link dataset of the training dataset. The training dataset was pre-processed for DBSCAN. Unnecessary data, marked as noise, were deleted from the training dataset. The DBSCAN-processed training dataset was augmented and sent to the CNN classifier (deep learning classifier). The test RSSI value was further converted into a 16 × 16 image and matched with previously trained datasets. The location of the matched image was the location of the test file.

**Figure 1:**
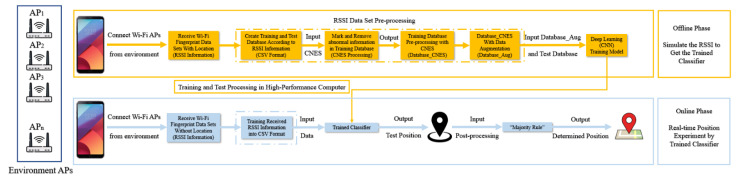
Fingerprint positioning with a deep learning classifier from [15].

In our previous paper [14], an indoor positioning technology framework based on deep learning classifiers was proposed as shown in Figure 1. In the paper, the positioning system framework was described as two phases: the offline phase and the online phase. The offline phase primarily involves the collection and processing of indoor positioning data. For example, the RSSI data are collected in the test environment, the resulting database is established and trained, and deep learning classifiers are trained. The result at this stage will directly affect the actual positioning accuracy. The online phase primarily involves the actual test, where the real-time positioning test is performed through the deep learning classifier obtained in the previous stage. Although the use of deep learning can improve the accuracy of indoor positioning, deep learning has not been effectively used due to insufficient database capacity. Therefore, we proposed a deep learning indoor positioning framework based on data augmentation in another manuscript [15]. In the offline phase, the program used data augmentation to increase the capacity of the RSSI fingerprint database to improve the training effect of the deep learning model. Experiments demonstrated that this method can further improve the positioning accuracy. In the last step of the online phase, the “majority rule” was used to select the most frequent positioning results returned by the server. This method is termed as the data post-processing algorithm [16]. This method can reduce the error in real-time positioning results to improve the positioning accuracy.

A previous study [17,18] showed that in a Wi-Fi fingerprint-based indoor positioning system, changes in a dynamic environment such as the multipath propagation of signals caused by obstacles near the user’s location, fading, and the addition or removal of Wi-Fi APs affect the indoor positioning accuracy. Furthermore, another study [19] noted that for an indoor positioning system in a dynamic environment, the error caused by the dynamic environment should be reduced through appropriate methods. Meanwhile, the latest research shows that the interference of moving objects and co-frequency interference in a dynamic environment may cause the Wi-Fi signal pattern to be changed over time, which reduces the positioning accuracy [20]. This paper presents a database pre-processing method based on a clustering-based noise elimination scheme (CNES) to effectively improve the real time positioning accuracy. The proposed CNES scheme is based on the DBSCAN method, and clustering and noise reduction processing were performed for the RSSI fingerprint data at each RP. The pre-processing of RSSI data with a clustering-based noise elimination scheme (CNES) is a novel concept. The proposed method successfully achieved the highest lab simulated positioning accuracy of 92.01% and a real time testing experimental positioning accuracy of 90.42%, which was much higher than the accuracy of the dataset without CNES pre-processing. Furthermore, the proposed method is an infrastructure-free method that does not require any additional infrastructure for implementation. The remainder of this paper is organized as follows. Section 2 presents the background; Section 3 discusses the proposed CNES data pre-processing scheme; Section 4 describes the numerical analysis and presents the laboratory simulation and experiments results; and finally, Section 5 summarizes the conclusions.

## 2. Background

### 2.1. Environment Setup

**Figure 2:**
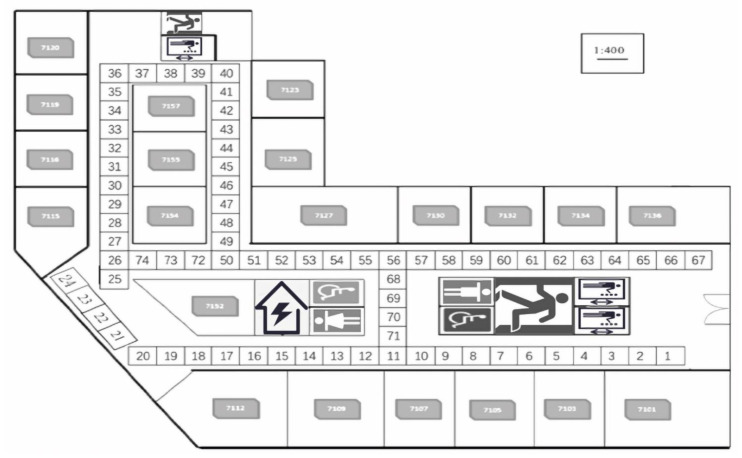
Environment setup for a floor map with 74 reference points.

Both data collection and experiment were performed on the seventh floor of the new engineering building at Dongguk University, Seoul, Korea. In Figure 2, the target area with the size of 52 m × 32 m and the roof height of 3 m is divided into 74 grids with 2 m × 2 m squares as the RP. Since each RP was assumed to be the center of the grid, any point in the grid was regarded as the RP. That is, the distance between any two adjacent RPs was considered as 2 m. The RPs such as 1, 25, 36, 40, and 67 were at the corner and their sizes varied between 2 m to 3 m (i.e., +1 m difference). Meanwhile, the RPs such as 10, 11, 18, 50, and 71 were at the ending spot and their sizes varied between 1 m to 2 m (i.e., −1 m difference). The positioning server used in this study was a Dell Alienware Model P31E (Alienware, hardware subsidiary of Dell, Miami, FL, USA), and the smartphone for data collection and testing was a Samsung SHV-E310K (chip fabrication Yongin-si, Gyeonggi-do, Korea). The fingerprint database construction, classification (i.e., position prediction), and online experimental setup were developed with Python. 

**Figure 3:**
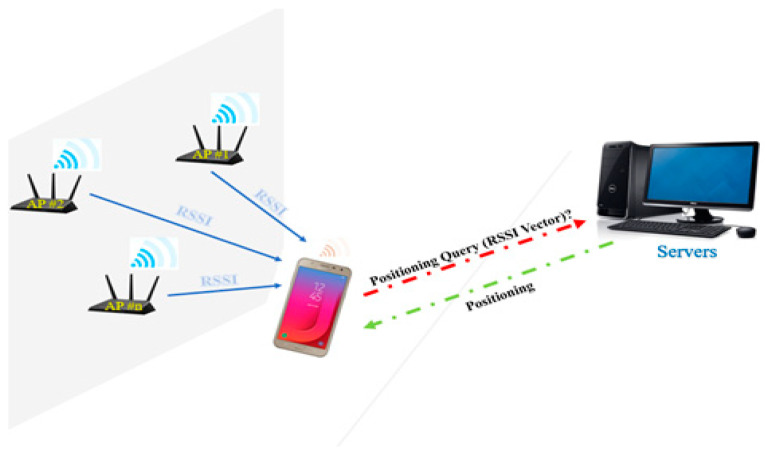
The proposed fingerprint-based Wi-Fi positioning system.

The data read by an Android device were stored in a buffer. If there was an error in the recorded data, an error message was displayed on a serially connected console. Otherwise, the RSSI data were stored in the buffer, and after a complete scan, they were transferred by the Android console, which was connected by an interface cable to the server, to the server through a Wi-Fi AP. The server determines the Android device’s location by comparing the measured RSSI values with the reference data. It was serially connected to the Android console and processed the RSSIs obtained from the surrounding APs with its CPU (Figure 3). The operating frequency of the Wi-Fi device was 2.412–2.480 GHz for the 802.11 bgn wireless standard. Additionally, the input/output sensitivity was 15–93 dBm.

### 2.2. CNN Model and Data Augmentation

**Figure 4:**
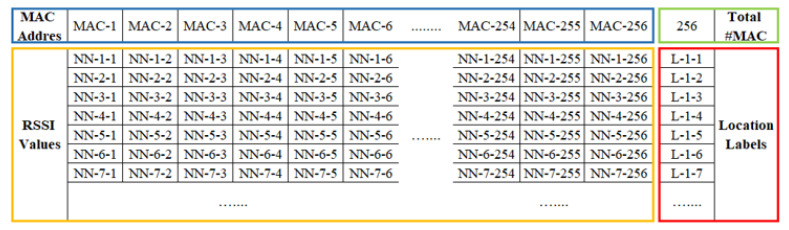
Input comma-separated-value (CSV) file format [14].

The collected RSSI data were converted into a comma-separated-value (CSV) file and then forwarded to the deep learning model. The structure of the generated CSV file is shown in Figure 4. The CSV file contained all the acquired RSSI information including the media access control (MAC) address from different APs, the RSSI value corresponding to each RP, and the number of RPs. The blue box shows the MAC address information area, which contained a total of 256 MAC information. 

**Figure 5:**
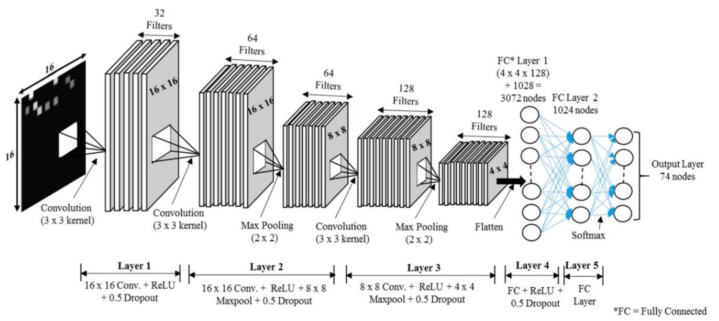
The convolutional neural network (CNN) architecture used in this study [14]. The second layer had a total of 18,496 parameters and the FC layer had 3072 counters, which led to the next hidden FC layer with 1024 converters. Finally, a softmax layer with 74 routines was used.

**Figure 6:**
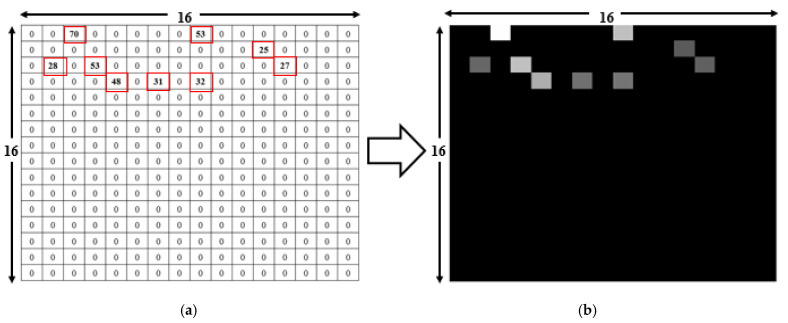
Deep learning input file conversion from a CSV file to an image. (**a**) Input CSV readings of the nine visible RSSIs from a total of 256 APs. (**b**) Converted grayscale image with nine bright spots representing APs visible at the RP [14].

The CNN classifier described in Figure 5 was proposed in our previous study [14], which was composed of five layers. The first layer had input grayscale images of size 16 × 16 × 1, rectified linear unit (ReLU), and dropout. Due to the small size of the input data set, max pooling was not used in the first layer. The second layer consisted of a 16 × 16 convolution with ReLU and then an 8 × 8 max pooling layer with a total of 18,496 parameters, which produced the output for the third layer with an 8 × 8 convolution with ReLU and then a 4 × 4 max pooling layer. The output was fed directly to a fully connected (FC) layer with 3072 nodes, which led to the next hidden FC layer with 1024 nodes. Finally, the output was calculated using a softmax layer with 74 nodes, which was the total number of RPs in our setup. The inner width was 1024, and the dropout of 0.5 was used for the first four layers. The learning rate was 0.001. The total number of parameters was 2,266,698. This calculated output was the total number of RPs in the current setup. The purpose of data enhancement is to obtain more training data by effectively transforming the existing data, thereby reducing the problem of under-fitting or over-fitting caused by the data quality or the amount of data being too small [21]. The input image was generated from the RSSI values received at 74 RPs during the experiment. At each RP, the RSSI value was recorded for 256 APs, though only a small subset of these APs was visible at each RP. Then, the RSSI values from different APs created a 16 × 16 image. For example, in Figure 6a, there are a total of nine visible RSSI values between 25 to 70 from 256 APs, with the other values of 0. As shown in Figure 6b, the RSSI values were converted into a grayscale image. The image had different levels of brightness depending on the recorded RSSI values, with higher RSSI values being brighter. The highest RSSI value was 70, which produced the brightest spot in the grayscale image, while the lowest value was 25, which is represented as the darkest nonblack spot. The RSSI values of 0 produced no brightness, so the remaining 247 spots were black. Similarly, the input RSSI files at the other 73 RPs produced different images as an input to the deep learning network.

Before providing the training data to the CNN model, we performed data augmentation for the training database by using the method presented in [21]. The augmentation scheme was operated using only the RSSI values collected at each RP. The RSSI value at each RP was randomly selected and written in a new CSV, which resulted in a large data size compared to the original CSV. The robustness lies in the fact that the pattern of augmented data well mimicked that of the RSSI data before augmentation. For the 24 dataset with 8880 images for each RP after augmentation, the total number of images at each RP was 532,800 with the size of 350 MB. Total number of test files was 1480.

### 2.3. RSSI Dataset Generation

To collect the RSSI data, we used a smartphone in the user’s hand and collected data five times on each RP. Each measurement comprised the RP label, the time, the date, the number of available APs, the MAC address, and the corresponding RSSI value at each RP. The RSSI measurement may contain noise, which seriously affects the positioning accuracy due to the time-varying channel characteristics. Moreover, indoor electromagnetic environments are complex and are characterized by multipath fading and other noise. We examined the RSSI fluctuation effects on prediction accuracy in [22]. In this work, the different directions (forward/backward) and times (morning/afternoon) were considered for seven-day data collection. A specific data collection procedure is as follows. The collector holds the smartphone at their waist position (about 1.2 m to 1.3 m height from the ground) and measures the data as stationary at each RP. In the morning, we conducted forward and backward data collections, respectively, which were repeated in the afternoon. Forward refers to the direction to collect the RSSI values from RP1 to RP74 in sequence. Meanwhile, backward means the opposite direction to collect the RSSI value from RP74 to RP1 sequentially. For a seven-day data collection, we collected 28 data files.

Table 1 shows the dataset types. The data collected in the morning and afternoon are denoted by M and A, respectively. The data collected in the forward and backward directions are labelled with F and B, respectively. The number represents the day number when the data were collected, as shown in Table 2. We divided 28 datasets into two parts. One part contained 24 datasets to construct the training database. The other part contained the remaining four datasets to build the test database. The RSSI values were measured five times at each RP in the forward as well as in backward directions. The sampling time for each RSSI measurement was 5 s, which was a total of 25 s for a total of five measurements. For 74 reference points, the total time consumed was 31 min in each direction and 62 min in both directions. For the training data, the measurements were made in the morning and the afternoon for seven days, which resulted in 15 h approximately. For the trial data, the measurements were made for two days in the same manner. 

## 3. Proposed Scheme

### 3.1. Density-Based Spatial Clustering of Applications with Noise (DBSCAN)

**Figure 7:**
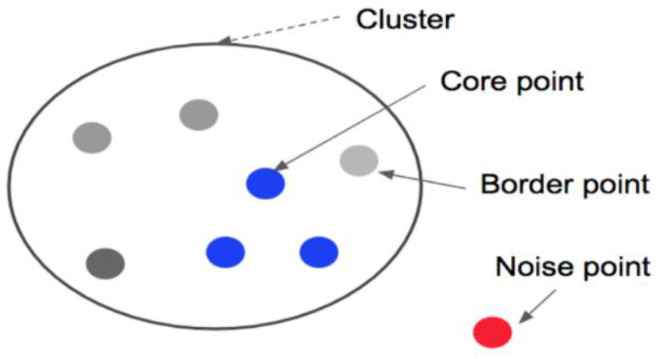
The concept of core points, border points, and noise points.

Previous approaches to indoor positioning technology focused on the study of small positioning areas. This was because it is necessary for noise samples to appear in the original data as the positioning area expands, especially in a dynamic environment. It has been confirmed [23] that the existence of noise would reduce the waste of computing resources and thus affect the accuracy of indoor positioning. Several studies have [22,23,24,25] used a clustering algorithm to cluster and divide the RSSI samples. These studies demonstrated that clustering could reduce the impact of noise in large-area positioning experiments. Density-based spatial clustering of applications with noise (DBSCAN) is a type of clustering that is well-known to the public. It is mainly based on the density of data, and it is highly representative. In this idea, a cluster is a large set, and all objects in it may be densely connected. The algorithm can be unconstrained in the sample database and is able to find clusters of any shape, which are major advantages. The DBSCAN process may be expressed concisely. In simple terms, the core point of a given dataset may be determined arbitrarily. Clustering around this point, all points with reachable density were included in the core point cluster. If many data have not been included, then re-clustering around a new core point is repeated in the cluster. Given a sample dataset, circle the given object with eps radius and count the data objects in this circle. Figure 7 uses a two-dimensional point set to illustrate the concept of core points, border points, and noise points. If there is a point with MinPts or greater in the eps radius around it, the other points will gather around that point, which is called the core point. Points that belong to a cluster but are not core points are called boundary points, and they are primarily points that form the outer edge of the cluster. Points that do not belong to any class become noise points.

### 3.2. Proposed Clustering-Based Noise Elimination Scheme (CNES)

**Figure 8:**
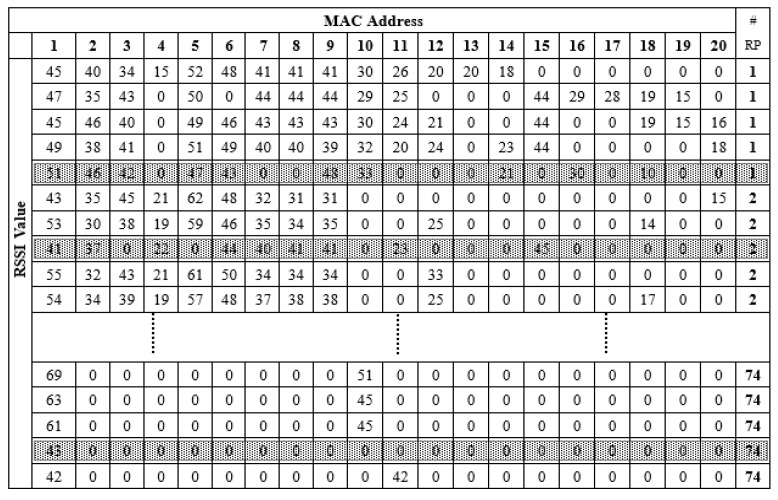
Clustering-based noise elimination scheme (CNES)-based training dataset with the highlighted and deleted noise points.

This paper proposed the CNES algorithm based on DBSCAN. The purity of the database was improved by detecting and deleting the noise points of each RP. The algorithm was run in the offline phase of the indoor positioning framework. After using CNES, the training database achieved the results shown in Figure 8. We performed the analysis of effective MinPts value and epsilon ‘eps’ of DBSAN for RSSI data noise elimination. The effective suitable MinPts value was 4. The value of eps could then be chosen by using a k-distance graph and plotting the distance to the k = minpts − 1 nearest neighbors ordered from the largest to smallest value. Furthermore, the best eps value was analyzed between 60 to 75 points. These points were further used to generate the final eps value.

**Figure 9:**
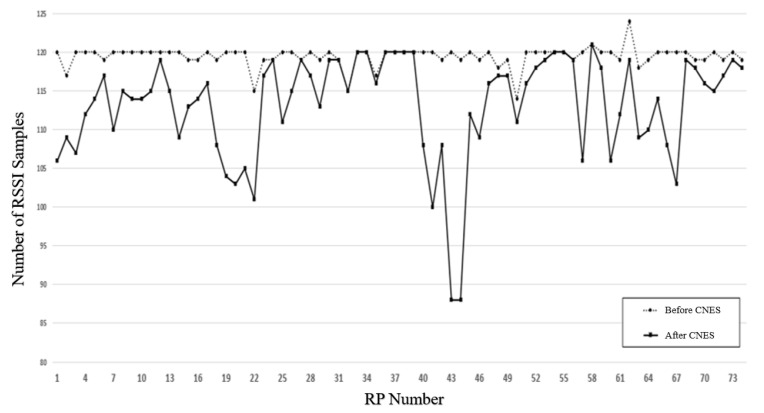
The effect of CNES corresponding to eps = 70 and MinPts = 4 on total number of RSSI samples at each RP.

**Figure 10:**
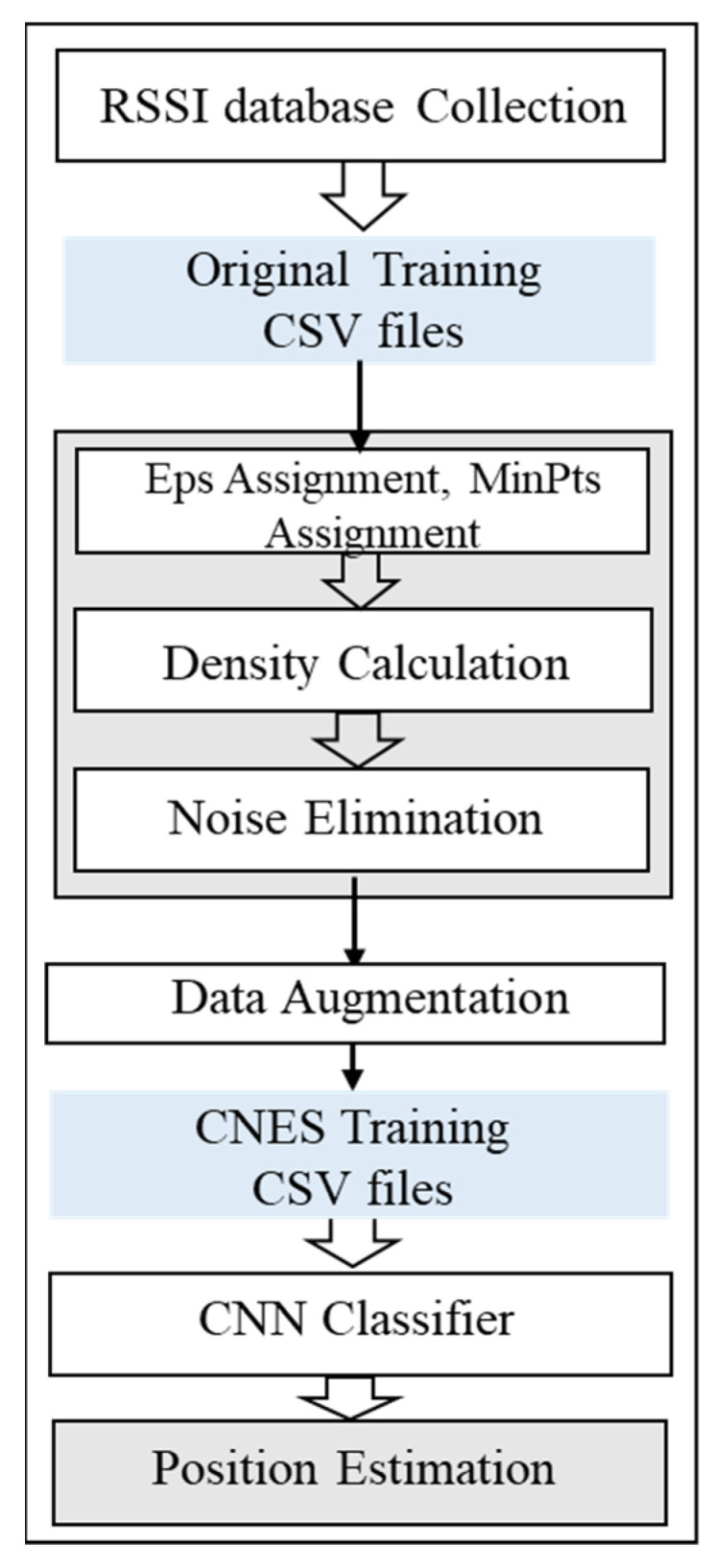
Flow graph for clustering-based noise elimination and position estimation.

Figure 8 shows the RSSI values of MAC addresses ranging from 1 to 20, taking the five sets of RSSI data samples for RP1, RP2, and RP74 as examples. The RSSI sample was the original CSV file generated from RSSI data collected in the experimental environment, which contained noise samples. When CNES is not used, direct use of the database will reduce the accuracy of indoor positioning. The grey, highlighted segments in Figure 8 represent noise samples such as the fifth group of RP1 samples, the third group of RP2 samples, and the fourth group of RP74 samples. The database was first imported into the CNES algorithm for processing; the processing marked and removed noise samples. Post-processing, the new database without noise samples was created. Finally, the new database processed data augmentation and deep learning model training and testing. Figure 9 shows the effect of CNES for eps = 70 and MinPts = 4. The dotted line represents the number of RSSI samples collected at each RP. The solid line represents the number RSSI samples after CNES for eps = 70 and MinPts = 4 at each RP. Figure 10 presents the complete flow graph for the proposed CNES database position estimation. In addition, the pseudocode of the proposed scheme is shown in Algorithm 1.
**Algorithm 1:** Pseudocode for Clustering-Based Noise Elimination and Position Estimation
1.  **Input:** Original CSV fingerprint training dataset 2.  **Define**: CSV dataset: 3.   **for** density calculation 4.    **Define** eps; minpts; 5.     **for** each reference point calculate density ‘D’ 6.      **if** RP == core point;    \\ Keep the RP data; 7.       **elseif** RP == edge point;   \\ Keep the RP data; 8.       **else** RP ≠ core point || RP ≠ edge point;  \\ Delete the RP data; 9.       **end if** 10.     **end for** 11.    **end for** 12.    Generate new CSV with density-based noise elimination point; 13.    Augment the output CSV file; 14.    Train the CNN classifier with new CSV file; 15.    Test the file for real time online position estimation; 16.    **end for**

## 4. Numerical Results

**Figure 11:**
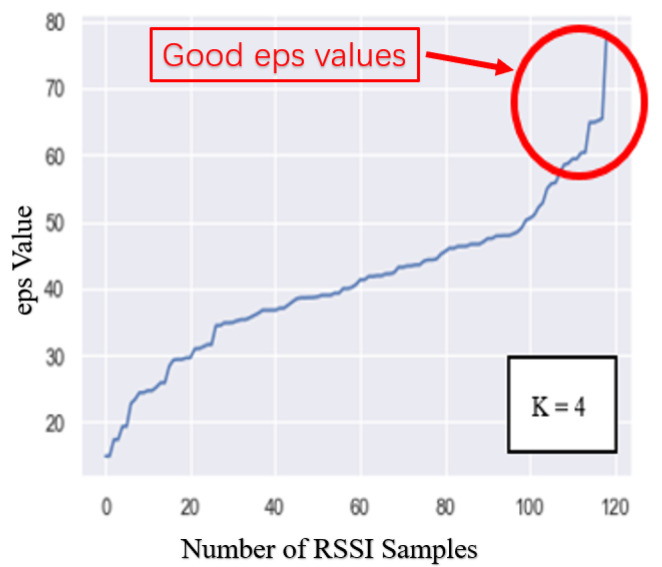
K-nearest neighbor distances to determine eps MinPts for CNES. The red circle represents “elbow”, which means there exist good eps values.

A total of 74 RPs was arranged in the positioning environment, as shown in Figure 2. Due to the dynamic environment, uncontrollable factors such as changes in the number of routers, the activation of telecommunication equipment and the movement of pedestrians can generate abnormal information such as noise in the collected RSSI information. DBSCAN recognizes the impact of noise and is robust to outliers. In the experiment, DBSCAN cluster analysis was performed for each RP point to reduce the influence of noise on the data during the data collection process, thereby improving the positioning accuracy. RSSI information was collected five times at each RP point, and a total of 24 datasets were collected in the experiment. Therefore, each RP point had 5 × 24 kinds of information, as shown in Figure 4. Then, for the total training set (comprising 74 RPs), there were a total of 74 fixed clusters because of the 74 RP labels. However, for each RP, the clustering algorithm marked and eliminated abnormal information, and therefore, there were two clusters for each RP. In the experiment, DBSCAN was used to cluster each RP point. This is because each RP was tagged when collecting RSSI information. The value of eps can be chosen by using a k-distance graph and plotting the distance to the k = minpts − 1 nearest neighbors ordered from the largest to the smallest value. Good eps values exist where the plot shows an ‘elbow’ (i.e., the threshold value above which the number of RSSI samples remains approximately the same), as shown in red circle in Figure 11. For example, eps = 70 in Figure 11. In general, for the suitable eps, a rule of thumb is to select the eps number with only a small fraction of RSSI samples.

### 4.1. Analysis of Eps

As mentioned, in order to find the best eps value, eps = {60: 75} was used to cluster the training database, and the database was then input into the deep learning model for indoor positioning simulation. In order to accurately verify the simulation accuracy corresponding to different eps values, when performing indoor positioning simulation, we chose the maximum positioning error acceptable in our indoor positioning system for analysis. Assuming the error distance was 4 m, the objective was to choose the eps value most suitable for our indoor positioning system. The simulation results are shown in Table 3, and the indoor positioning accuracy was as high as 94.191% when eps = 70.

**Figure 12:**
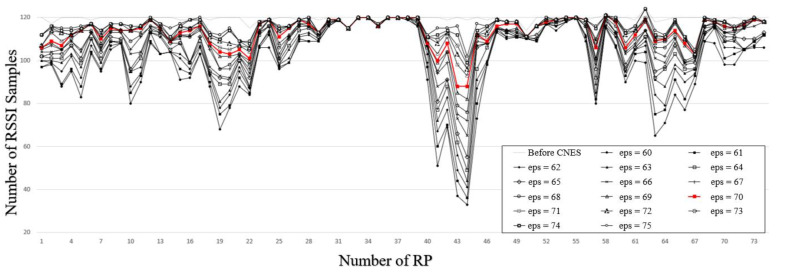
The effect of CNES on the number of RSSI samples at each RP corresponding to different eps values.

In the experiment, DBSCAN was used to cluster the RSSI samples of each RP in the training database, and the RSSI samples outside the core point neighborhood could be eliminated by using the best eps value. The eliminated samples were also the so-called errors. Information samples were not suitable for positioning reference information. Therefore, in the training set clustered by different eps values, the number of RSSI samples retained by each RP was inconsistent, as shown in Figure 12. Among the lines, the top dashed line represents the original training set, that is, with all original RSSI samples retained. As the eps value increases, the curve approaches closer to the original curve. At RP = [10, 11, 19, 20, 21, 22, 41, 42, 43, 44, 45, 56, 64, 65, 66, 67], the range of change becomes larger. In particular, at the point RP = [41, 43, 44], the range of change exceeded 70. This is because these points show the areas where the Wi-Fi signals and people were dense, which may cause RSSI degradation. The red line in Figure 12 denotes eps = 70, which was the best eps value in Table 3. For eps = 70, the average number of removed samples was six, which was lower than 10 for all eps values, which means that more samples were removed in Figure 12. Furthermore, for eps = 70, it was shown that all reference samples were retained at RP = [32, 33, 36, 37, 38, 54, 55, 58] when the environment was better less than the external interference.

### 4.2. Lab Simulation Results

In the CNN model, the lab simulation results with the highest accuracy were selected for real time testing. In terms of the accuracy of both models, the lab simulation results are shown in Table 4. 

When the RP number is accurately predicted by the CNN trained model, it is called Margin-0 (i.e., 0 m error). When the predicted test RP matches the neighboring RP, it is called Margin-1 (i.e., 2 m error). Similarly, when the test RP matched with difference of two RPs, it is known as Margin 2 (i.e., 4 m error). A comparison of the accuracies of the CNN mode for different margins and for the two techniques is presented in Table 4. As shown in Table 4, the CNES scheme can improve positioning accuracy. Without CNES, the positioning accuracy of Margin-0 was 43.50% only. At the same time, Margin-1 was 75.95%, and Margin-2 was 87.26%. However, the positioning accuracy was significantly improved after using the CNES scheme. The positioning accuracy of Margin-0 exceeded 60%, which was 61.28%. In this way, the positioning effect of Margins-1 and -2 using the CNES solution was also obvious. In particular, the simulation result of Margin-1 reached 83.15%, which was close to the result of Margin-2 without CNES. In addition, the result of Margin-2 exceeded 92%. In addition, we compared the difference between with and without CNES under the same margin. The simulation positioning accuracy after using CNES was improved by 17.78% (Margin-0), 7.24% (Margin-1), and 4.75% (Margin-2). Through a comparison, it can be seen that CNES can significantly improve the accuracy of indoor positioning. In terms of Margin-0, the improvement effect was significant, which means that indoor positioning using CNES can achieve an error of zero-meters in most cases.

**Figure 13:**
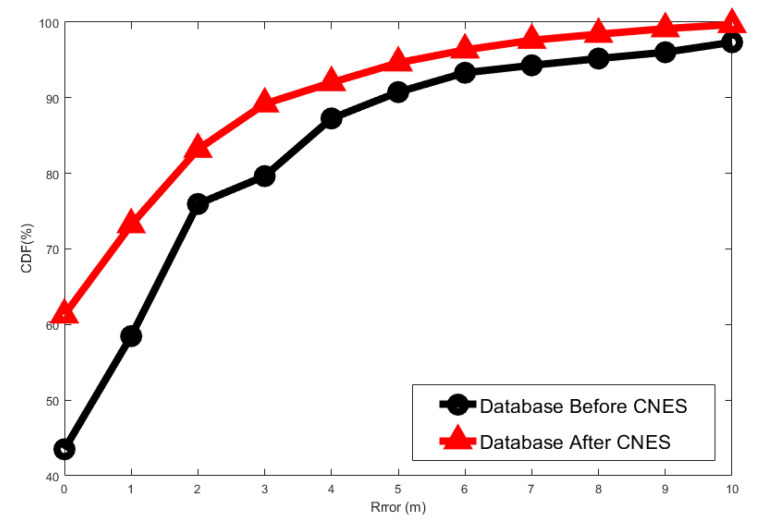
Cumulative distribution function (cdf) vs. distance error. *X*-axis represents positioning errors and *Y*-axis represents the positioning accuracy for different positioning errors.

The effectiveness of the proposed CNES was also evaluated in terms of positioning accuracy, which is defined as the cumulative distribution function (cdf) of the location error within a specified distance in Figure 13. It was shown that the CNES outperformed that without CNES over the entire range. Note that when the cdf exceeded 94%, the distance error with the CNES was only 4.76 m. Meanwhile, the distance error without CNES was 6.70 m. In addition, it was shown that the cdf with CNES was 73.21% for a one-meter error, but that without CNES was only 58.47%.

### 4.3. PCA

**Figure 14:**
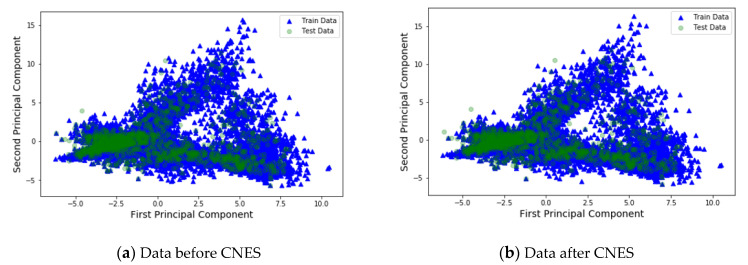
Principal component analysis (PCA) plots for two schemes: (**a**) before and (**b**) after applying the CNES. Green points are test data, and blue points are training data.

Principal component analysis (PCA) diagrams for the training database and test database for the five cases are shown in Figure 14, and the blue points in the figure represent the RPs in the training data without data augmentation, and the green points represent the unknown location points, that is, the points in the test data. From Figure 14, (a) represents the analysis of data before CNES was applied, and (b) represents the analysis of data after CNES was applied. Furthermore, it is evident that after the CNES was applied, the RP points in the training data were more compact than those in the training data to which the CNES was not applied. This is because the discrete points in Figure 14a are caused by incorrect RSSI fingerprint information, and the use of the CNES reduces wrong information in the training set, thereby improving the accuracy of the deep learning simulation.

### 4.4. Experimental Results with Real Time Testing

In the experiments, we used the trained classifier for real time testing. The specific process involves passing the measured RSSI values at RPs into the CNN model to obtain the features. Then, the obtained features were compared with those in the trained classifier. The RP with the most similar features was determined as the final position. In addition, we compared two cases for real time testing, namely, CNN and CNES + CNN. “CNN” means CNN without CNES, and “CNES + CNN” denotes CNN with CNES. For real time testing, we made experiments four times with the plan in Table 5. The experiments were made for two working days (D-1 and D-2), and four times (Test 1, 2, 3, and 4) a day.

When processing the experimental results, we stipulated that the test results with measurement errors less than or equal to 2 were successful, which means that only the positioning result and the current position error distance of less than four meters can be used, and the data represent the probability of successful results obtained in the measurement results. An example of the experimental results is shown in Table 6. As shown in Table 6, for each RP positioning test in an experiment, five positioning decisions were performed continuously for the same RP.

In order to facilitate the comparison of the experimental results of CNES, we merged all experimental results into Table 7. Meanwhile, the results were rearranged and are shown in Table 8. It can be seen that the CNES scheme can effectively improve the indoor positioning accuracy, especially in the case of Margin-0 (zero-meter error), the average positioning success rate increased by 22.43%. Without the CNES scheme, the Margin-0 (zero-meter error) positioning success rate was only 39.45%, and after using the CNES scheme, the Margin-0 (zero-meter error) positioning success rate exceeded 60%, which was 61.88%. Meanwhile, the CNES scheme had outstanding positioning accuracy in Margin-1 (two-meter error), and the positioning success rate was 82.77, which was close to the positioning success rate in Margin-2 without the CNES scheme. In addition, in the case of Margin-2 (four-meter error), it exceeded the highest success rate of 90.42% without the CNES scheme. Therefore, the above data show that the CNES pre-processing scheme can indeed greatly improve the accuracy of indoor positioning without changing the hardware.

It is generally known that the multipath effect of the channel will increase the abnormal information (such as noise) in the RSSI dataset. Therefore, the abnormal information in the original training dataset can be eliminated by using the CNES scheme, and then the purity of the training dataset can be improved. According to the simulation and actual experiments, the databases with CNES can adapt to various databases and different environments, thereby improving the positioning accuracy.

## 5. Conclusions

In this work, a deep learning solution involving a clustering processing scheme was developed. The results showed that the use of pre-processed data along with the CNES could effectively improve the indoor positioning accuracy. The simulation results showed that when the CNES was used as the clustering algorithm, the best effect was obtained for eps = 70. For the indoor positioning simulation, with the CNES RSSI dataset, a positioning accuracy of 92.01% was achieved. The experimental results in the real environment also showed that the CNES pre-processing scheme could increase the positioning accuracy by 22.43%, 9.15%, and 5.21 in Margin-0 (zero-meter error), Margin-1 (two-meter error), and Margin-2 (four-meter error), respectively. Furthermore, the CNES scheme could reduce the effect of interference factors in the dynamic environment on the positioning accuracy and improve the adaptability of indoor positioning accuracy.

## Figures and Tables

**Table 1:** Database information and augmentation size.

Database Type	Collection	# of Images	
		Before Augmentation	After Augmentation
Training	24 sets	8880	532,800
Test	4 sets	1480	--

**Table 2:** Types of dataset.

Dataset	Forward	Backward	Number of Data Files
Morning	MF1, MF2, ..., MF7	MB1, MB2, ..., MB7	14
Afternoon	AF1, AF2, ..., AF7	AB1, AB2, ..., AB7	14
Number of Data Files	14	14	28

**Table 3:** Simulation accuracies for different eps values (training epochs number = 1000, MinPts = 4).

Eps Value	Lab Simulation Accuracy	Eps Value	Lab Simulation Accuracy
60	93.594%	68	93.491%
61	93.193%	69	92.889%
62	93.293%	70	94.191%
63	92.789%	71	93.189%
64	93.889%	72	92.893%
65	92.593%	73	92.292%
66	92.490%	74	93.093%

**Table 4:** Summary of lab simulation results.

Lab Simulation Model	Margin (%)		
	0	1	2
CNN	43.50	75.95	87.26
CNES + CNN	61.28	83.19	92.01
Difference	17.78	7.24	4.75

**Table 5:** The experimental plan for real-time testing.

Day	Test 1	Test 2	Test 3	Test 4
D-1	CNN	CNES + CNN	CNN	CNES + CNN
D-2	CNES + CNN	CNN	CNES + CNN	CNN

**Table 6:** The example of real time testing experimental results.

RF #	Positioning Decision #					# of Success Decisions		
	#1	#2	#3	#4	#5	Margin-0	Margin-1	Margin-2
1	1	1	2	1	1	4	5	5
2	2	3	2	4	2	3	4	5
3	3	3	2	3	4	3	5	5
••••••								
72	72	73	73	74	72	2	4	5
73	73	73	73	73	26	4	4	5
74	73	74	74	26	26	2	4	5
Experiment Success Rate (%)						61.71	83.35	91.62

**Table 7:** Summary of real time testing of experimental results.

Day	Database (Test Number)	Margin			Database Test Number)	Margin		
		0	1	2		0	1	2
D-1	CNN (Test 1)	38.97	73.78	85.13	CNN (Test 3)	39.12	72.95	84.81
	CNES + CNN (Test 2)	61.71	83.35	91.62	CNES + CNN (Test 4)	62.28	82.47	89.69
D-2	CNES + CNN (Test 1)	62.03	82.73	90.11	CNES + CNN (Test 3)	61.48	82.51	90.27
	CNN (Test 2)	40.23	74.06	85.79	CNN (Test 4)	39.47	73.69	85.14

**Table 8:** Difference of experimental average results (%).

Database	Average Margin		
	0	1	2
CNN	39.45	73.62	85.22
CNES + CNN	61.88	82.77	90.42
Difference	22.43	9.15	5.21

## Data Availability

Not applicable.
